# Thrombotic Thrombocytopenic Purpura due to Checkpoint Inhibitors

**DOI:** 10.1155/2018/2464619

**Published:** 2018-12-20

**Authors:** Alexey Youssef, Nawara Kasso, Antonio Sergio Torloni, Michael Stanek, Tomislav Dragovich, Mark Gimbel, Fade Mahmoud

**Affiliations:** ^1^Centre for Tropical Medicine and Global Health, Nuffield Department of Medicine, University of Oxford, Oxford, UK; ^2^Faculty of Medicine, Tishreen University, Lattakia, Syria; ^3^Medical Director, Stem Cell Therapy, Apheresis, and Transfusion Medicine, Banner MD Anderson Cancer Center, Gilbert, AZ, USA; ^4^Division of Hematology, Banner MD Anderson Cancer Center, Gilbert, AZ, USA; ^5^Chief, Division of Medical Oncology and Hematology, Banner MD Anderson Cancer Center, Gilbert, AZ, USA; ^6^The T.W. Lewis Melanoma Center of Excellence, Banner MD Anderson Cancer Center, Gilbert, AZ, USA

## Abstract

Ipilimumab is a monoclonal antibody that enhances the efficacy of the immune system by targeting a cytotoxic T-lymphocyte-associated protein 4 (CTLA-4), which is a protein receptor that downregulates the immune system. Nivolumab is also a humanized monoclonal antibody that targets another protein receptor that prevents activated T cells from attacking the cancer; this receptor is called programmed cell death 1 (PD-1). The FDA approved ipilimumab combined with nivolumab as a frontline therapy for patients with metastatic melanoma or renal cell carcinoma and as a second-line therapy for patients with microsatellite instability-high (MSI-H) metastatic colon cancer. Immune-related adverse events such as autoimmune colitis, pneumonitis, hepatitis, nephritis, hypophysitis, and thyroiditis may occur during or weeks to months after therapy. We report a case of thrombotic thrombocytopenic purpura (TTP) in a patient with metastatic renal cell carcinoma following one cycle of ipilimumab and nivolumab. Only one case report of ipilimumab-induced TTP exists in the medical literature. With the wide use of immunotherapy to treat cancers, physicians need to be aware of this rare immune-related adverse event.

## 1. Introduction

Thrombotic thrombocytopenic purpura (TTP) is a thrombotic microangiopathy (TMA) characterized by thrombocytopenia due to platelet consumption, hemolytic anemia due to red blood cell (RBC) fragmentation, and organ damage due to blood flow blockage by clots that are high in platelets with little or no fibrin [1]. TTP is caused by the deficiency of a circulating, von Willebrand factor (vWF) cleaving metalloprotease, ADAMTS13 (A Disintegrin And Metalloprotease with ThromboSpondin-1 motif, member 13) [2, 3]. The lack of ADAMTS13 can be either familial (a mutation in the ADAMTS13 gene) or acquired (through inhibitory antibodies generated against ADAMTS13). TTP is diagnosed by a severe deficiency of ADAMTS13 activity of less than 10% [1–3]. Here, we present a case of TTP following administration of ipilimumab and nivolumab.

## 2. Case Presentation

A 42-year-old woman was admitted to our hospital with change in mental status, slurred speech, and fever 9 days following the administration of one cycle of the intravenous infusion of ipilimumab 1 mg/kg and nivolumab 3 mg/kg for the treatment of metastatic renal cell carcinoma (RCC). Her history is significant for a right radical nephrectomy with lymphadenectomy for a kidney mass identified on CT abdomen and pelvis during the work up of hematuria (January 2018). Pathology confirmed papillary RCC with extensive sarcomatoid features. Eight of the 11 lymph nodes were involved with cancer. She received Sutent (50 mg orally daily, 4 weeks on and 2 weeks off) for 4 months and then stopped due to progression of disease. Spine MRI revealed a C3 compression deformity with tumor extension as well as osteolytic metastatic disease at C4 and the right C5 pedicle. She underwent C2 to C5 posterolateral arthrodesis and instrumentation. Ipilimumab and nivolumab were initiated on 06/04/2018. Four days later, she presented to clinic with significant fatigue. Laboratory results revealed hemoglobin (Hb) 4.9 g/dL; hematocrit (HCT) 16.4%; and platelets (PLT) 36,000 per microliter. She was given prednisone 1 mg/kg orally daily for presumed immunotherapy-induced immune thrombocytopenia (ITP) and received 2 units of packed RBCs. Her overall health deteriorated so she got admitted on 06/13/2018. Laboratory studies on admission are available in Table 1.

MRI brain revealed calvarial metastasis but no evidence of intracranial disease. Electroencephalogram (EEG) revealed moderate generalized disturbance in the cerebral slowing activity. A diagnosis of TTP was made on the basis of laboratory and clinical findings. Methylprednisolone 125 mg IV every 6 hours, therapeutic plasma exchange (TPE: 1 to 1.5 plasma volumes per treatment; a total of 8 treatments; exchange fluid of 5% human albumin and FFP), and rituximab (weekly 4 doses) were initiated. ADAMTS13 activity less than 3% and inhibitory titer 9.9 Bethesda Units/mL confirmed acquired TTP. Ten days after initiating the appropriate therapy, the mental status improved, the platelet count increased to 116,000 per microliter, and the LDH level decreased to 406 U/L.

## 3. Discussion

TTP was once identified by a clinical pentad: thrombocytopenia, microangiopathic hemolytic anemia, neurologic symptoms, renal function abnormalities, and fever. However, this pentad is a clinically rare finding in patients with TTP. Thrombocytopenia and microangiopathic hemolytic anemia remain the most consistent signs of TTP [2]. Neurologic symptoms are the most common, ranging from headaches to seizures. D-dimer, indicative of fibrinolysis and thrombin activation, is usually normal or mildly elevated in patients with TTP. Increased plasma fibrinogen and D-dimer levels are associated with increased tumor size, higher nuclear grade, advanced TNM stage, and poor survival in patients with RCC [4]. Our patient did not have a concomitant DIC; hence, increased D-dimer is likely due to advanced RCC. ADAMTS13 activity less than 10% is diagnostic for TTP. TTP is an emergency that requires immediate intervention. The keystone in TTP management is TPE; the typical goal is to exchange 1 to 1.5 times the estimated plasma volume per treatment until a stable LDH and a platelet count more than 100k per microliter is reached. TPE is tapered after that [2]. The autoimmune nature of TTP rationalizes the use of corticosteroids as an adjuvant treatment to TTP [1, 2]. Another important agent in the treatment of TTP is rituximab, which is a monoclonal antibody that targets CD20 receptors on B-cell surface [5]. Research implies that rituximab is effective in unresponsive cases of acquired TTP. Early use of rituximab has also improved the control of TTP; this has prompted the inclusion of rituximab as a frontline treatment [1, 5].

Ipilimumab is a monoclonal antibody that binds to cytotoxic T-lymphocyte-associated antigen 4 (CTLA-4). By blocking inhibitory signals transmitted by CTLA-4, ipilimumab reactivates cytotoxic T-lymphocytes (CTLs) and enables them to attack cancer cells [6]. Ipilimumab is used in combination with nivolumab to treat advanced melanoma and non-small cell lung carcinoma [7]. Recently, the list of indications for the aforementioned combination has been expanded by the FDA to include advanced renal cell carcinoma (RCC) [8]. Nivolumab is another immune checkpoint inhibitor. Cancer cells make PD-L1 (programmed cell death ligand 1) which binds to PD-1 (programmed cell death 1) receptors on T cells and deactivates them. Nivolumab is a monoclonal anti-PD-1 antibody that helps T cells to maintain their activity and to clear cancer cells [7]. Immunotherapy is known to cause immune-related adverse events (irAEs). Such adverse events may be organ specific, such as colitis, hepatitis, and hypothyroidism, or general like fatigue and rash [9]. The combination of ipilimumab and nivolumab offers a more efficient treatment for advanced malignancies, albeit a more toxic one. Research has implied that the majority of patients treated with this combination have developed irAEs and most of which were responsive to proper management [10]. In contrast to ipilimumab, anti-PD-1 antibodies cause less prevalent and less serious irAEs [10]. Our patient developed laboratory-confirmed immune-mediated TTP shortly after receiving ipilimumab and nivolumab. To the best of our knowledge, there is only one paper reporting TTP after ipilimumab [11]. We acknowledge that advanced malignancy could cause TTP. In most cancer-related TTP cases, TTP is detected at the initial diagnosis of cancer. However, in about 20% of cases, it is seen at the time of cancer recurrence and usually reflects the late stage of disease [12]. Mechanisms that may account for cancer-related TTP include direct invasion of the bone marrow by cancer cells which damage the endothelial cells of the marrow vessels leading to the release of ultralarge vWF multimers, formation of ADAMTS13 autoantibodies, and disturbance of the vascular endothelial cells by mucin [12].

**Figure 1:**
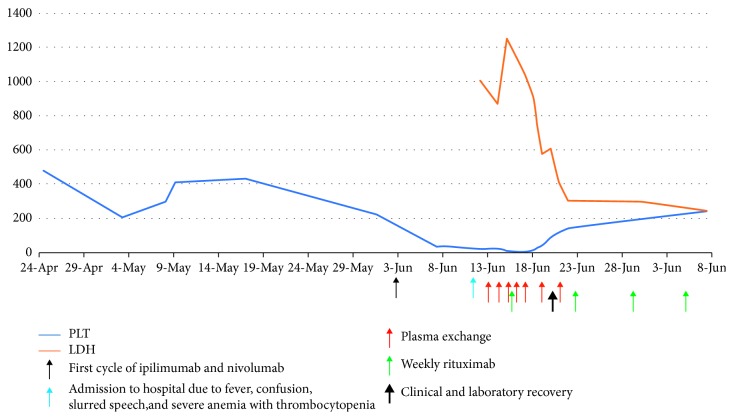
Clinical, laboratory, therapeutic, and recovery course.

The platelet count of our patient was stable prior to the administration of ipilimumab and nivolumab, despite the aggressive and advanced nature of her disease.  Figure 1 depicts a sudden drop in the platelet count along with a high level of LDH. These two findings coincide with the initiation of ipilimumab and nivolumab therapy. Moreover, the cancer-related TTP would only get better by treating the underlying malignancy. Follow-up body CT scans, in our patient, revealed further progression of disease which indicated that her TTP should have gotten worse, not better in case it was cancer-related TTP. The fact that her mental status improved and platelet count and LDH levels decreased indicates that the underlying etiology of her TTP is likely due to immunotherapy. Using the Naranjo algorithm [13], we assessed the probability of an adverse drug reaction (ADR). In our case, the Naranjo algorithm resulted in a score of +4; this translates clinically into a possible ADR. There is a previous conclusive report of TTP after ipilimumab (+1); the adverse event appeared after ipilimumab was given (+2); the adverse event improved when the drug was discontinued (+1); the reaction was confirmed by objective laboratory measurements (+1). However, the event might have been caused by cancer (−1). The exact mechanism underlying checkpoint inhibitors-induced TTP remains unknown. Several CD4 + T-cell epitopes of ADAMTS13 in HLA DRB1∗11 and HLA-DRB1∗3 patients have recently been identified. The major B-cell epitope resides in the spacer domain, whereas DRB1∗11 and DRB1∗03 presented peptides reside in the CUB2 domain of ADAMTS13. These epitopes are responsible for the immunodominant response to ADAMTS13. It is hypothesized that FINVAPHAR and ASYILIRD CD4+ T cells are involved in the onset and/or relapse of acquired TTP [14, 15]. As TTP is a rarely reported irAEs of checkpoint inhibitors, early recognition of this rare presentation is essential for timely and appropriate management.

## Figures and Tables

**Table 1:** Laboratory studies on admission.

Variable	On admission
Hgb	6 g/dL
HCT	18.7%
PLT	20,000 per microliter
LDH	998 U/L
Total bilirubin	1.9 mg/dL
Creatinine	0.9 mg/dL
D-dimer	7342 ng/mL
Haptoglobin	<10 mg/dL
Fibrinogen	349 mg/dL
PT	12 sec
INR	1
PTT	31 sec
Heparin-induced anti-PF4 antibodies	0.312
Direct Coomb's test	Negative
Blood culture	Negative
Urine culture	Negative
Peripheral blood smear	Fragmented erythrocytes Polychromasia Nucleated RBCs Schistocytes
